# Increased expression of interleukin-6 gene in gastritis and gastric cancer

**DOI:** 10.1590/1414-431X2020e10687

**Published:** 2021-05-17

**Authors:** M.P. Santos, J.N. Pereira, R.W. Delabio, M.A.C. Smith, S.L.M. Payão, L.C. Carneiro, M.S. Barbosa, L.T. Rasmussen

**Affiliations:** 1 Laboratório de Genética, Faculdade de Medicina de Marília, Marília, SP, Brasil; 2 Departamento de Morfologia, Universidade Federal de São Paulo, São Paulo, SP, Brasil; 3 Instituto de Patologia Tropical e Saúde Pública, Universidade Federal de Goiás, Goiânia, GO, Brasil

**Keywords:** Interleukin 6, Interleukin 6-receptor, Helicobacter pylori, Gastric cancer, Polymorphisms

## Abstract

*Helicobacter pylori (H. pylori)* induces an intense inflammatory response, mediated by proinflammatory cytokines, including interleukin (IL)-6 and its membrane receptor (IL-6R), which activates important signaling pathways in the development of gastric disease and cancer. We investigated the gene and protein expression of *IL-6* and *IL-6R* and the influence of polymorphisms rs1800795, rs1800796, and rs1800797 on its gene expression together with *H. pylori* infection. Furthermore, an *in-silico* analysis was performed to support our results. Gastric biopsies were obtained from patients with gastric symptoms and patients with gastric cancer (GC) and were divided into groups (Control, Gastritis, and Cancer). *H. pylori* was detected by PCR. Real-time-qPCR was employed to determine gene expression, and western blot assay was used to analyze protein expression levels. PCR-RFLP was used to characterize *IL-6* polymorphisms. Bioinformatics analyses were performed using the Gene Expression Omnibus (GEO) database and GEO2R to screen out differentially expressed genes (DEGs). *H. pylori* was detected in 43.3% of the samples. Statistically significant differences were found for *IL-6* (P=0.0001) and *IL-6R* (P=0.0005) genes among the three groups, regardless of the presence of *H. pylori*. Among patients with *H. pylori* infection, the *IL-6* and *IL-6R* gene and protein expressions were significantly increased, highlighting *IL-6* gene overexpression in patients with GC. No statistically significant differences were found for the rs1800795, rs1800796, and rs1800797 polymorphisms compared to *IL-6* gene expression. The results indicated that the *IL-6* polymorphisms do not influence its expression, but *IL-6* and *IL-6R* expression seems to be altered by the presence of *H. pylori.*

## Introduction

Since its first identification in 1983 by Robin Warren and Berry Marshal, *Helicobacter pylori* (*H. pylori*) has been associated with many gastric diseases, including gastric cancer (GC) (1). GC is the fourth most common type of cancer in the world and the second leading cause of cancer death (2). The interaction of *H. pylori* with the gastric epithelium promotes an inflammatory response in the gastric mucosa, which is mediated by proinflammatory cytokines, such as interleukin 6 (IL-6). Physiologically, IL-6 is involved in the defense of the organism, functioning as a messenger between an innate and adaptive immune response, and the presence of *H. pylori* promotes an increase in its synthesis, contributing to inflammation and leading to gastritis (3,4).

*IL-6* gene can regulate tumor growth and indirectly promote the growth of tumor cells, by inducing apoptosis in leukocytes (5). However, to develop its functions in the biological control of processes, such as B cell differentiation, apoptosis, and cell proliferation, its membrane receptor (*IL-6R*) must be present. The *IL-6/IL-6R* complex activates important signaling pathways in inflammatory and carcinogenic processes (6–9). When expressed, it may act as an anti-apoptotic factor in esophageal carcinoma cells by activating the STAT3 and JAK signaling pathway (7). Taniguchi and Karin (10) showed that increased expression of *IL-6* gene favored the invasion of cells from carcinoma in the esophagus. Similarly, a significant association was observed between high expression of *IL-6R* gene and tumor invasions and metastases (7).

Moreover, *IL-6* gene expression may also be influenced by the presence of polymorphisms such as rs1800795, rs1800796, and rs1800797 in the promoter‐region of the gene, and it may promote changes in its functional activity (11). According to Jurečekova et al. (12), the exchange of a guanine for a cytosine in *IL-6* gene (rs1800795) seems to have an influence and may result in altered expression and functional activity of *IL-6*, suggesting that this exchange is carried out with the transcription and expression of the gene. The study conducted by Lippitz and Harris (13) found a significant association between the polymorphism rs1800796 and the risk of cancer, considering the G allele as a risk factor. The polymorphism rs1800797 is associated with several diseases, including increased risk of developing cancer. However, the results are still controversial and inconclusive (14).

As previously described, the expression of *IL-6* gene might be influenced by the presence of polymorphic sites in the gene and may be involved in the process of GC development and the immune response.

There are few studies in the literature that simultaneously analyzed and associated gene expression with protein expression, polymorphisms of clinical importance, and the presence of *H. pylori*. We understand that this association may play a key role in the etiology and development of gastric cancer. Therefore, this study investigated the gene and protein expressions of *IL-6* and *IL-6R* as well as the influence of the polymorphisms rs1800795, rs1800796, and rs1800797 on *IL-6* and *IL-6R* gene expression, considering the presence of *H. pylori* and developing GC.

## Material and Methods

### Patients and samples

This study was performed with 254 samples from patients with peptic symptoms. Three biopsies from the antrum were taken from each patient: one biopsy was used for DNA extraction, one for RNA extraction, and another for histological analysis. Then, the groups were divided into Control, Gastritis, and Cancer according to histological analysis based on the Sydney system and the Lauren's classification (15). The groups consisted of 139 samples from patients with chronic gastritis (82 women/57 men mean age: 54±17 years), 64 samples from patients without lesions in the gastric mucosa (Control group) (41 women/23 men, mean age: 55±15 years), and 51 samples from patients with gastric adenocarcinoma (31 women/20 men, mean age: 53±9 years).

Age and sex did not differ statistically among the groups studied (P>0.05). The ethnic origins were self-declared and consisted of 90% European, 2.5% Japanese, and 7.5% of mixed origin. The authors understand that the ethnic data had limited strength.

The State Hospital of Bauru and the Gastroenterology Center of the Clinical Hospital of Marilia Medical School collaborated to obtain the gastric biopsies. The Federal University of São Paulo and the Federal University of Goias collaborated with samples from GC patients. Patients who received anti-inflammatory or antibiotic treatment were excluded from the study. The Ethics and Research Committee of Universidade do Sagrado Coração approved this study (case number 1.119.830) and all subjects signed a statement of consent to participate in the study.

### RNA and DNA extraction and *Helicobacter pylori* detection

All gastric antrum tissue samples used for DNA and RNA extraction were stored in RNAlater (Ambion, USA) at −20°C. For RNA extraction, approximately 40 mg of tissue were homogenized in Precellys 24 tissue homogenizer (Bertin Corp., USA), and RNA was isolated using a miRNeasy Mini Kit (Qiagen, Germany, cat. No. 217004) according to the manufacturer's instructions. RNA concentrations were determined and adjusted using the Nanodrop 2000 spectrophotometer (Thermo Scientific, USA).

DNA extraction was carried out according to the protocol established for the Qiagen QiaAmp Kit (Qiagen, cat. No. 51304).

*H. pylori* was detected using the polymerase chain reaction (PCR) technique, using the Hpx1 (CTGGAGARACTAAGYCCTCC) and Hpx2 (GAGGAATACTCATTGCGAAGGCGA) oligonucleotide pair, which amplified a 150-bp fragment, under the conditions of 40 cycles of 1 min at 94°C, 1 min at 59°C, and 1 min at 72°C. After amplification, the fragments were visualized on 1.5% agarose gel (16).

### Genotyping of *IL-6* polymorphisms

To confirm the presence of the rs1800795, rs1800796, and rs1800797 polymorphisms, the PCR-RFLP technique was performed using specific oligonucleotides for each polymorphism (Table 1) (17). The amplified fragments were treated with specific enzymes also described in Table 1 and then visualized on 2.5% agarose gel. The description of the amplified products and the expected fragments are described in previous research (18).

**Table 1:** Oligonucleotides used to amplify the fragments in question and their restriction enzymes.

Position	Sequence (5′- 3′)	Amplicon	Enzymes	Reference
rs1800795	F 5′ TTGTCAAGACATGCCAAAGTG 3′	300pb	*Nla*III	Ogilvie et al. (17)
	R 5′ TCAGACATCTCCAGTCCTATA 3′			
rs1800796	F 5′ AGATTCCAAGGGTCACTTG 3′	279pb	*Bsr*BI	Developed in the laboratory
	R 5′ AGAAGCAGAACCACTCTTC 3′			
rs1800797	F 5′ AGATTCCAAGGGTCACTTG 3′	297pb	*Bse*GI	Developed in the laboratory
	R 5′ AGAAGCAGAACCACTCTTC 3′			

### cDNA synthesis and real-time quantitative PCR (qPCR)

For the synthesis of complementary DNA (cDNA), only samples with a ratio value between 1.85 and 2.2 were used. cDNA was synthesized using a High-Capacity cDNA Reverse Transcription kit (Applied Biosystems™, USA) and following the manufacturer's protocol.

The qPCR was performed on the ABI Prism 7500 Fast Sequence Detection System equipment, using taqman: *IL-6* (Hs00174131_m1) and *IL-6R* (Hs01075664_m1) (the target genes), and *UBC* (Hs00221499_m1) and *TBP* (Hs00187332_m1) (the reference genes). The relative quantification of the expression was calculated by the 2^-ΔΔ^Ct method, according to Livak and Schmittgen (19).

### Bioinformatics analysis

#### Screening for differentially expressed genes (DEGs)

To support our results, we also performed an *in silico* analysis to identify DEGs using three gene expression profiles.

In the Gene Expression Omnibus (GEO) Profiles database (https://www.ncbi.nlm.nih.gov/gds), we used the keywords “gastric cancer and Interleukin 6” and got 1.956 results. Then, we did a careful review and selected three expression profiles in the GEO database: GSE2685, GSE27411, and GSE4651.

GSE2685 (30 human samples: 22 gastric cancer samples and 8 controls) was based on the GPL80 platform [Hu6800] Affymetrix Human Full Length HuGeneFL Array and the expression profiling of 22 primary advanced gastric cancer tissues was compared with normal gastric mucosa. GSE27411 (9 human samples), based on the GPL6255 platform Illumina humanRef-8 v2.0 expression beadchip, was used to compare tissue infected with *H. pylori* and uninfected tissue. The dataset GSE4651 (5 *Mus musculus* samples) from the GPL6246 platform [MoGene-1_0-st] Affymetrix Mouse Gene 1.0 ST Array [transcript (gene) version] was used to analyze differentiated expression in transgenic animals 6 months following infection with *Helicobacter felis.*

### Western blot assay

The total protein was extracted from samples of gastric biopsies using RIPA buffer containing inhibitors (1 M NaF, Complete Protease Cocktail Inhibitor, and 0.1 M PMSF). The total extracted proteins were quantified using the NanoDrop 2000 spectrophotometer (Thermo Scientific^®^) using Bradford reagent and standard BSA proteins (5000201, Bio-Rad, USA). The proteins (25 μg) were separated in 12% SDS-PAGE, and transferred to a nitrocellulose membrane, and then blocked with 5% skimmed milk in 1× phosphate buffered saline and 0.1% Tween 20 (PBST), for 2 h at room temperature. Subsequently, the membranes were incubated overnight at 4°C, with primary antibodies: anti-α-tubulin antibody (5168, 1:5,000, Santa Cruz Biotechnology, USA); anti-IL-6 (93356, 1:500, Abcam, USA), and anti-IL-6R (128008, 1:3,000, Abcam). Then, the membranes were incubated individually for 1 h 30 min at room temperature with secondary antibodies: goat anti-rabbit IgG H&L (97051, 1:10,000, Abcam), used for *IL-6* and *IL-6R*, and m-IgGk BP-HRP (1:5.000 Santa Cruz Biotechnology), used for tubulin. Finally, the membranes were washed in PBST, and revealed.

### Statistical analyses

The analyses were performed using GraphPad Prism 5 (USA). ANOVA, two-tailed Student's *t*-test (and nonparametric tests), and Fisher's exact test were performed for statistical analyses. To determine differences in expression and elaborate volcano plots, adj.P.Val (adjusted by Benjamini & Hochbergand) and logFC were used. Results with P<0.05 were considered to be statistically significant.

## Results

### *Helicobacter pylori* detection

*H. pylori* was detected in 110/254 (43.3%) samples, with a higher prevalence in samples from patients with gastritis and GC. Our results indicated that the presence of *H. pylori* increased the risk for the development of gastric diseases. Details of the distribution of *H. pylori* in the groups studied as well as the results are described in Table 2.

**Table 2:** Frequency of *Helicobacter pylori* infection in Control, Gastritis, and Cancer groups.

	Control (n, %)	Gastritis (n, %)	Cancer (n, %)	Total
*H. pylori* +	12 (18.8)	62 (44.6)	36 (40.6)	110
*H. pylori* -	52 (81.2)	77 (55.4)	15 (29.4)	144
Total	64 (100)	139 (100)	51 (100)	254
OR (95%CI)		3.489 (1.713-7.107), 0.0003^A^	4.622 (1.799-11.880), 0.0016^B^	

^A^Control *vs* Gastritis; ^B^Control *vs* Cancer (Student’s *t*-test).

### *IL-6* and *IL-6R* gene expression

An initial analysis was performed by comparing the Control *vs* Gastritis *vs* Cancer groups, regardless of the presence of *H. pylori* for *IL-6* and *IL-6R*. Then, we performed a more refined analysis in which *H. pylori* presence was considered. For this, the groups were subdivided by considering infection (positive and negative) and were compared to each other and to the Control negative group (without gastritis and negative for *H. pylori*).

**Figure 1:**
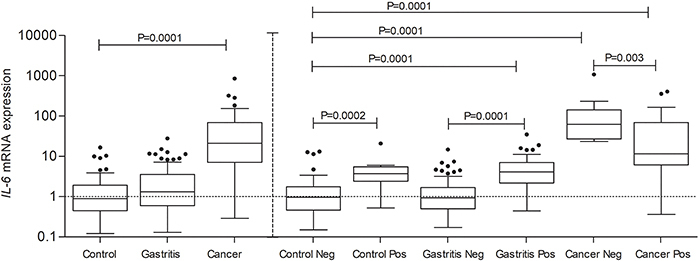
Analysis of interleukin (*IL-6*) expression (percent of Control) in Control, Gastritis, and Cancer groups when the presence of *H. pylori* was not considered (**left**), and when it was considered (**right**). Data are reported as medians and interquartile range (ANOVA). Control neg and pos: normal gastric tissue without gastritis or inflammatory process and negative or positive for *H. pylori* infection; Gastritis neg and pos: patients with gastritis and negative or positive for *H. pylori*; Cancer neg or pos: patients with cancer and negative or positive for *H. pylori*.

In the first analysis, statistically significant differences were found of *IL-6* (P=0.0001) and *IL-6R* (P=0.0005) genes, not considering the presence of the *H. pylori* bacterium. In the second analysis, considering the presence of *H. pylori,* similar results were found for both genes. An increased expression of *IL-6* was found in all the groups studied when comparing them to Control negative group, except for the Gastritis negative group (Figure 1).

**Figure 2:**
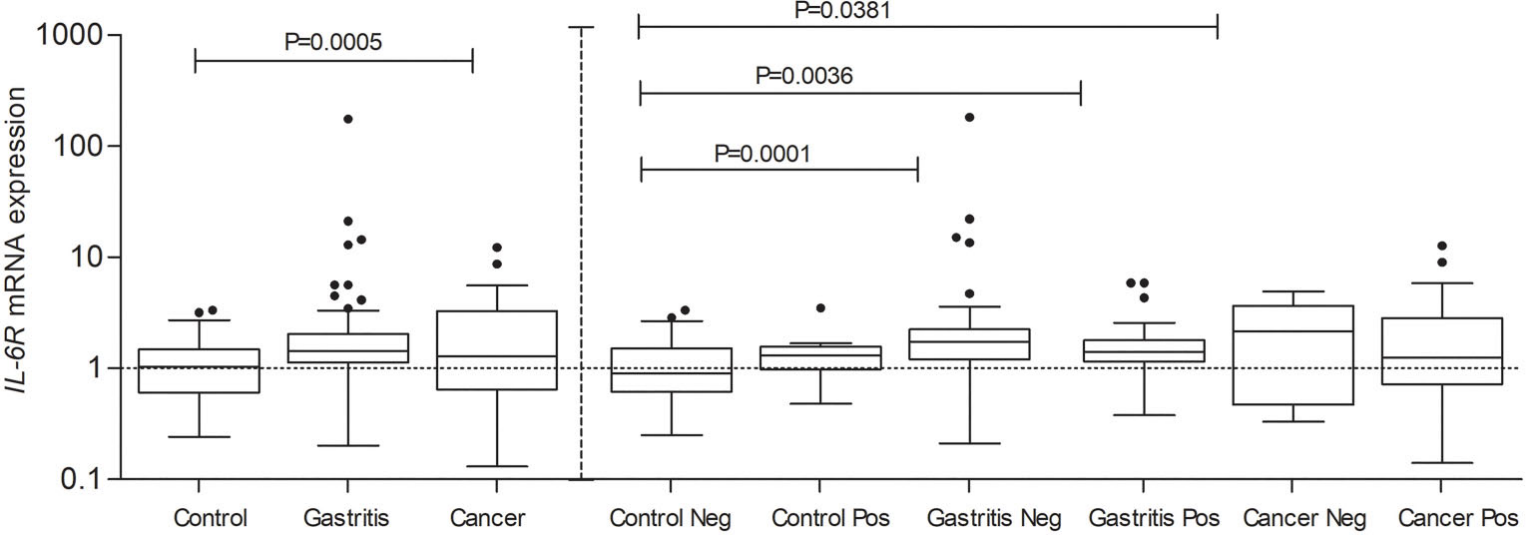
Analysis of interleukin-6 receptor (*IL-6R*) expression (percent of Control) in Control, Gastritis, and Cancer groups when the presence of *H. pylori* was not considered (**left**), and when it was considered (**right**). Data are reported as medians and interquartile range (ANOVA). Control neg and pos: normal gastric tissue without gastritis or inflammatory process and negative or positive for *H. pylori* infection; Gastritis neg and pos: patients with gastritis and negative or positive for *H. pylori*; Cancer neg or pos: patients with cancer and negative or positive for *H. pylori*.

Analysis of the *IL-6R* gene, considering the presence of *H. pylori,* found statistically significant differences in Gastritis negative, Gastritis positive, and Cancer negative groups, in relation to Control negative (Figure 2).

### *IL-6* gene expression and polymorphisms

**Figure 3:**
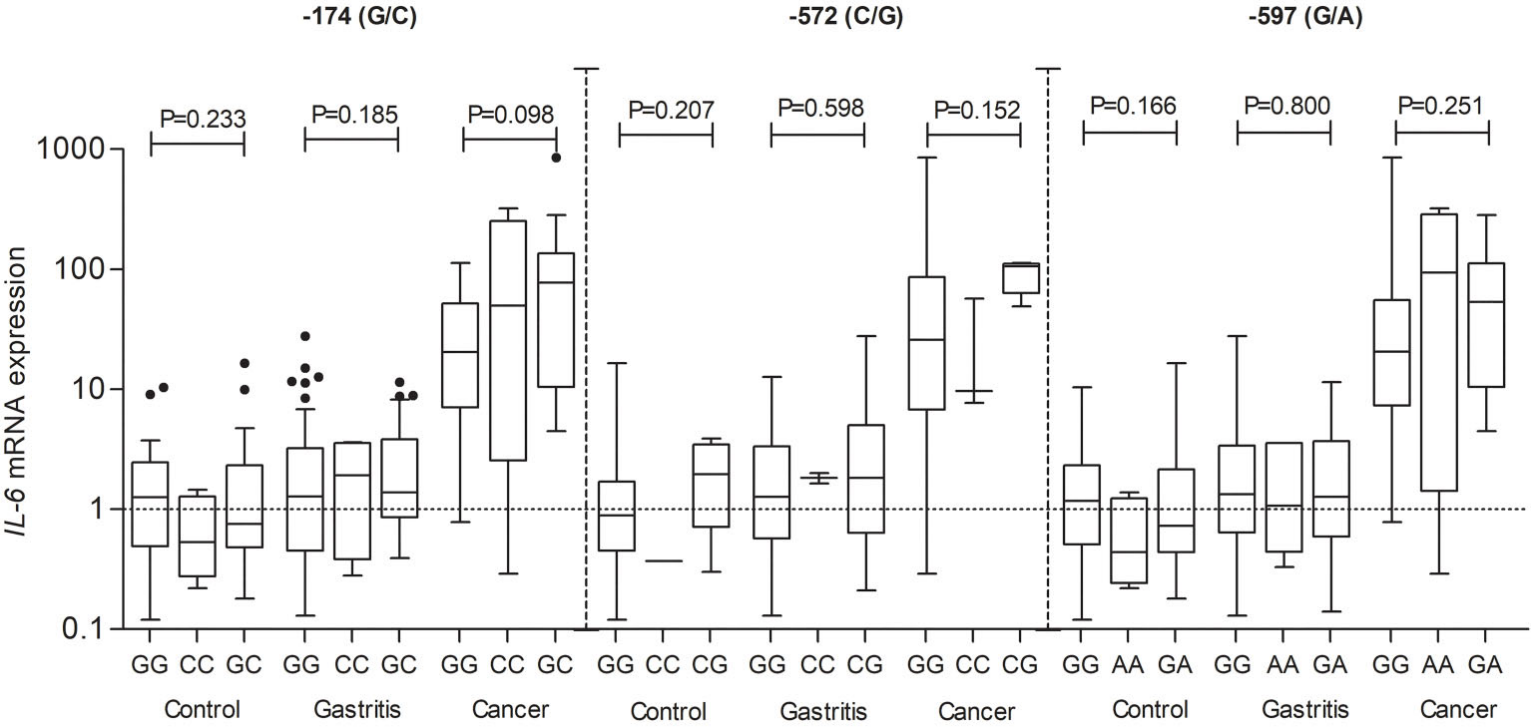
Analysis of interleukin-6 (*IL-6*) gene expression and rs1800795, rs1800796, and rs1800797 polymorphisms (percent of Control) in Control, Gastritis, and Cancer groups. Data are reported as medians and interquartile range (ANOVA).

At this point, the analyses for the *IL-6* gene expression were performed again. The genotypes of each polymorphism (rs1800795, rs1800796, and rs1800797) were considered in each group, and compared to *IL-6* gene expression, and no statistically significant differences were found for the three polymorphisms regardless of the group. Figure 3 shows *IL-6* gene expression levels and the polymorphisms in all groups.

### Identification of DEGs

**Figure 4:**
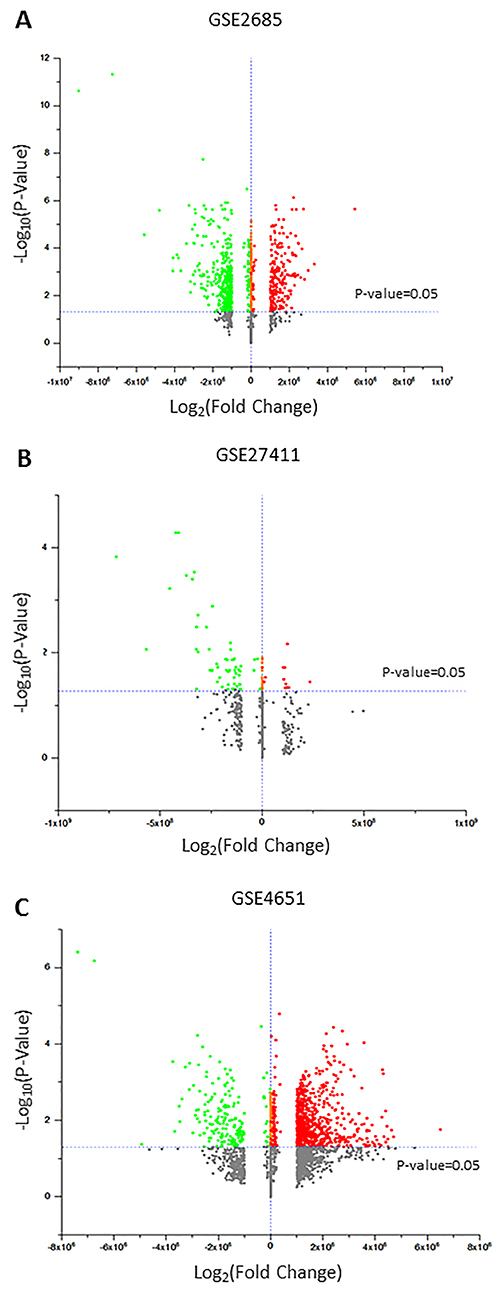
Differentially expressed genes (DEGs) between Gastric Cancer samples and Normal samples. **A**, The volcano plot for DEGs in GSE2685 data, **B**, in GSE27411 data, and **C**, in GSE4651 data. The red dots represent upregulated and the green dots represent downregulated genes, both screened on the basis of j-fold change j >1.0, and adjusted of P<0.05.

Three gene expression profiles GSE2685, GSE27411, and GSE4651 were selected and subjected to analysis. Considering the GSE2685, a total of 688 DEGs were obtained, of which 449 genes were downregulated and 239 genes were upregulated (Figure 4A). In the GSE27411 dataset, 68 genes were found (56 genes were downregulated and 12 genes were upregulated) (Figure 4B). Finally, in GSE4651, 1,015 were obtained (187 genes were downregulated and 828 genes were upregulated) (Figure 4C).

We would like to highlight that, among the genes with increased expression in GSE2685 (239 genes) and GSE4651 (828 genes) datasets, *IL-6* gene was located at positions 110 and 429, respectively, confirming the results obtained in our study. On the other hand, although *IL-6R* appears in the GSE2685 database, its expression is contrary to our results.

### IL-6 and IL-6R protein expression

**Figure 5:**
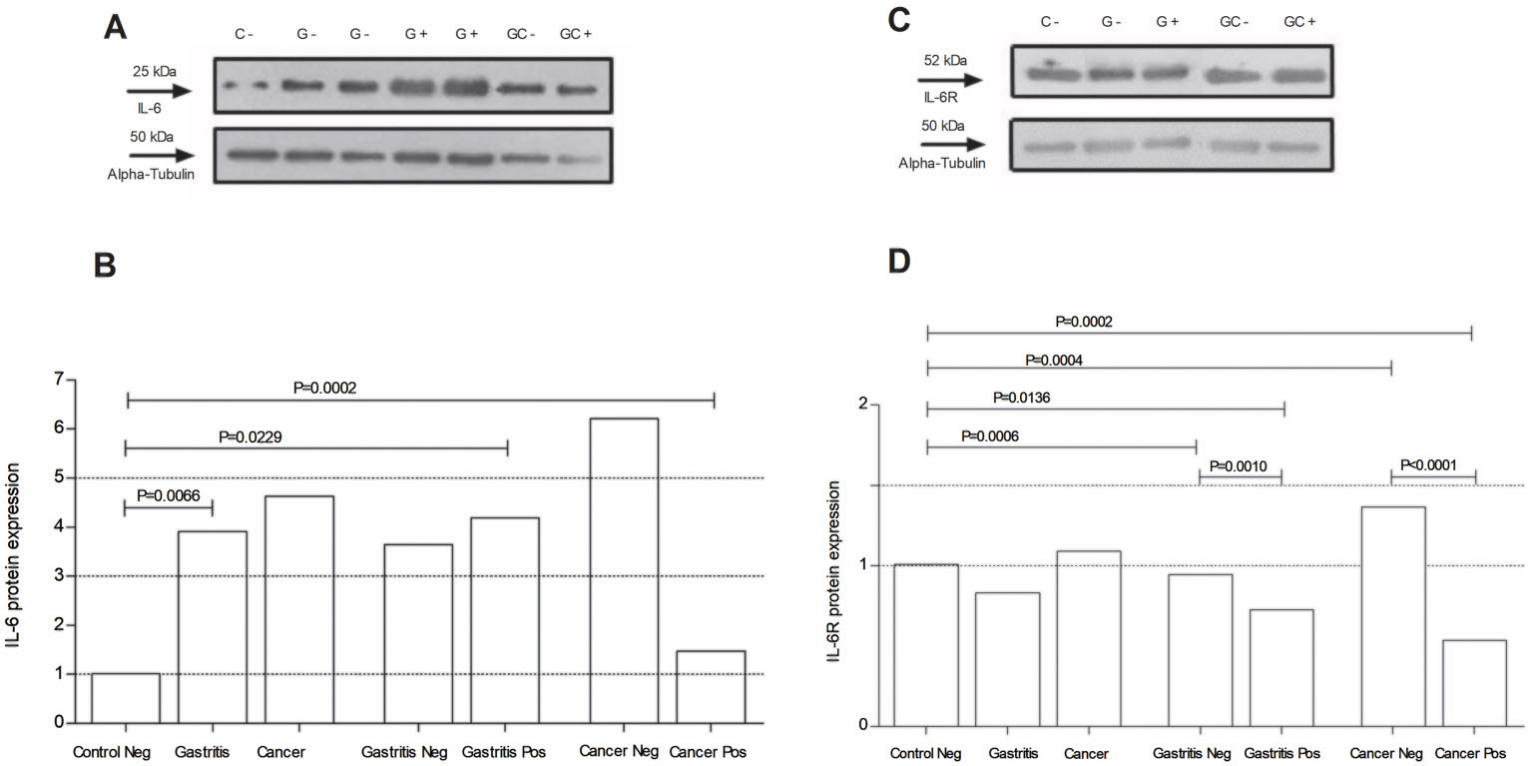
**A**, Levels of interleukin (IL)-6 were examined in samples from patients with Gastritis (G) and Gastric Cancer (GC) and from Control (C) by western blot analysis. **B**, Analysis of IL-6 protein expression in groups, considering or not the presence of *H. pylori*. **C** and **D**, Levels of IL-6 receptor (IL-6R) in the same samples. Data are reported as medians and interquartile range (Student's *t*-test). neg and pos: negative and positive for *H. pylori* infection.

To analyze the expression levels of IL-6 and IL-6R proteins, western blot technique was employed (Figure 5A-C). Similar to the analyses performed for gene expression, the groups were subdivided according to the presence or absence of *H. pylori*, and then compared to the negative Control group, for both proteins.

As for the expression levels of the IL-6 protein, regardless the presence of *H. pylori*, statistically significant differences were found in the comparison between the Control negative group and the Gastritis group (P=0.0066), as shown in Figure 5B. In the subsequent analyzes, in which *H. pylori* presence was considered, statistically significant differences were found in the groups Gastritis and Cancer, both positive for *H. pylori,* compared to the Control negative group (Figure 5B).

In relation to IL-6R protein, statistically significant differences were found for Gastritis negative and positive groups, and for Cancer negative and positive groups compared to Control negative group and between these groups (Figure 5D).

## Discussion

*H. pylori* has been reported as the most common cause for the development of GC, since its colonization in the gastric mucosa leads to an intense inflammatory process, stimulating the transcription and production of proinflammatory molecules, such as *IL-6*. In this study, *H. pylori* was detected in 43.3% of the samples analyzed, with a higher prevalence in the Gastritis and Cancer groups compared to the Control group, confirming that its presence increased the risk for the development of gastric diseases. These results are in agreement with other studies (20,21).

Several studies have explored the possible association between the presence of rs1800795, rs1800796, and rs1800797 polymorphisms and changes in *IL-6* gene expression, which appear to undergo transcriptional changes and influence the susceptibility of some diseases, including cancer (12,14,22).

*IL-6* gene has been extensively studied in the development of GC, because inflammation and cancer are directly connected (13,23). Inflammatory processes, such as that caused by *H. pylori* infection, increase the synthesis of IL-6, activating the immune response and signaling pathways, which may contribute to the development of tumors. JAK, STAT3, PI3K, MAPK, and AMPK are some of the signaling pathways activated by the IL-6 signaling cascade, which depend on the presence of the IL-6R, mainly expressed on the hepatocyte cell membrane, neutrophils, monocytes/macrophages, and some lymphocytes. This interaction between IL-6 and IL-6R stimulates cells, which express only gp130, in a process known as trans-signaling (9). Activation of these signaling pathways by IL-6/IL-6R justifies their important role in inflammation and the consequent development of cancer. Several studies have shown that the ability of *IL-6* to activate STAT3 results in increased expression of apoptosis and cell cycle-linked genes, in addition to regulating cell proliferation via the growth factor signaling pathway. IL-6 activation of STAT3 seems to influence vital processes of tissue homeostasis maintenance. Thus, *IL-6* gene regulates some of the key steps in controlling inflammation and establishes the anti-inflammatory environment (6–8). In this context, some studies have tried to understand how *IL-6* is capable of protecting and resolving inflammation, but can also cause several harmful consequences to the organism, especially in chronic and progressive diseases (24,25).

In this study, the mRNA and protein analyses revealed an increased expression of *IL-6* and *IL-6R* in patients with gastritis and cancer, especially when considering the presence of *H. pylori*. *In silico* analyses were performed considering GC samples to support our findings. In this case, *IL-6* appeared with increased expression in two of the three databases selected in this study, confirming our results. In agreement with our findings, Wu et al. (26) observed higher expression in neoplastic tissues than normal tissue.

In results that were consistent with ours, the expression of *IL-6* was related to the promotion of human GC invasion and migration of AGS cells. Immunohistochemistry and *in-situ* hybridization have shown that IL-6 is highly expressed and is secreted from both gastric epithelial cells and inflammatory cells infiltrating the gastric mucosa in *H. pylori* infection (23). Zuo et al. (27) obtained similar results, with greater expression in the cancer group than the control group. In another study, the findings demonstrate increased expression of *IL-6* in prostate cancer patients, also considering the presence of *H. pylori* (6). Furthermore, increased *IL-6* expression was found in recurrent tumors compared to primary tumors, as well as recurrent metastases (8).

Our findings were in agreement with those of Simondurairaj et al. (7) who observed high *IL-6R* expression in patients with GC and metastasis, which suggested that *IL-6* is capable of promoting neoplastic cell proliferation and invasion. The study by Jiang et al. (28) reported that inhibition of highly expressed *IL-6R* in neoplastic tissues led to a reduction in cell proliferation as well as an increase in apoptosis. In other words, the highly expressed *IL-6/IL-6R* complex in cancer patients is related to inhibition of cell apoptosis, and therefore related to a worse prognosis in these patients. However, our results on *IL-6* gene promoter indicated that polymorphisms did not influence their expression, although the results in the literature are controversial and inconclusive.

Jurečekova et al. (12) also observed an increase in *IL-6* mRNA expression but did not find any association between the presence of the rs1800795 polymorphism and the alterations of its gene expression. Nevertheless, Huang et al. (29) demonstrated that the presence of the rs1800796 polymorphism is able to modulate the transcriptional activity of the *IL-6* gene, resulting in a high risk of prostate cancer. The meta-analysis by Peng et al. (14) showed that the three polymorphisms influence the expression of *IL-6*, contributing to the progression of cancer.

Although most studies have not found an association of these polymorphisms with GC development, the study by Zhang et al. (5) offered a weak link between rs1800796 polymorphism for risk of this neoplasia and association in the male subgroup, indicating that susceptibility may be modified by gender. In disagreement, Hwang et al. (30) suggested, through *in vitro* analyses, that these polymorphisms are not able to influence the levels of *IL-6* used by human stomach cells, especially when stimulated by the presence of *H. pylori*. With results consistent with our study, Wang et al. (31) also found no association between polymorphisms and the development of GC.

Together, our results indicated that *H. pylori* was associated with an increased risk for the development of gastric diseases, as already described in the literature. Furthermore, we found an increase in *IL-6* and *IL-6R* gene and protein expression in patients with gastritis and GC, which seems to be influenced by the presence of *H. pylori*. However, we speculate that after neoplastic transformation, this correlation is lost and this overexpression of the *IL-6 gene* in neoplastic tissues may be a tumor progression strategy inducing the apoptosis in defense cells and favoring invasion and proliferation of cancer cells. Moreover, the *IL-6* gene polymorphisms (rs1800795, rs1800796, and rs1800797) were not associated with alterations in *IL-6* gene expression.
